# A reporter model to visualize imprinting stability at the *Dlk1* locus during mouse development and in pluripotent cells

**DOI:** 10.1242/dev.138255

**Published:** 2016-11-15

**Authors:** Emily Swanzey, Matthias Stadtfeld

**Affiliations:** The Helen L. and Martin S. Kimmel Center for Biology and Medicine, Skirball Institute of Biomolecular Medicine, Department of Cell Biology, NYU School of Medicine, New York, NY 10016, USA

**Keywords:** Genomic imprinting, Dlk1, Fluorescent reporter, Allele-specific expression, DNA methylation, Pluripotency

## Abstract

Genomic imprinting results in the monoallelic expression of genes that encode important regulators of growth and proliferation. Dysregulation of imprinted genes, such as those within the *Dlk1-Dio3* locus, is associated with developmental syndromes and specific diseases. Our ability to interrogate causes of imprinting instability has been hindered by the absence of suitable model systems. Here, we describe a *Dlk1* knock-in reporter mouse that enables single-cell visualization of allele-specific expression and prospective isolation of cells, simultaneously. We show that this ‘imprinting reporter mouse’ can be used to detect tissue-specific *Dlk1* expression patterns in developing embryos. We also apply this system to pluripotent cell culture and demonstrate that it faithfully indicates DNA methylation changes induced upon cellular reprogramming. Finally, the reporter system reveals the role of elevated oxygen levels in eroding imprinted *Dlk1* expression during prolonged culture and *in vitro* differentiation. The possibility to study allele-specific expression in different contexts makes our reporter system a useful tool to dissect the regulation of genomic imprinting in normal development and disease.

## Abstract (precis)

**Summary:** A *Dlk1* knock-in reporter mouse reports allele- and tissue-specific *Dlk1* expression in developing embryos that can be used to study changes in genomic imprinting during cellular reprogramming.

## INTRODUCTION

More than 100 mammalian genes are expressed in a predominantly monoallelic fashion in a paradigmatic epigenetic event referred to as genomic imprinting (Bartolomei and Ferguson-Smith, 2011). Imprinted genes, such as those within the commonly studied *Dlk1-Dio3* gene cluster, are regulated by gender-specific DNA methylation marks at imprinting control regions (ICRs) (Duffie and Bourc'his, 2013; Sanli and Feil, 2015). Failure to preserve allele-specific imprinted gene expression, such as by the acquisition of hypermethylation at the ICR (referred to as loss-of-imprinting or LOI), can have detrimental developmental consequences and is a hallmark of cancer (Peters, 2014). The factors that contribute to the epigenetic instability of imprinted genes remain largely elusive, partly because of the absence of suitable model systems. At present, imprinting is typically studied by assessing DNA methylation levels or nucleotide polymorphisms in imprinted transcripts. These tools, however, are restricted to either bulk populations (Babak et al., 2015; Hammoud et al., 2013) and/or retrospective analysis (Ginart et al., 2016).

*Dlk1* is a paternally expressed protein-coding gene within *Dlk1-Dio3* (da Rocha et al., 2008) that regulates fetal growth. We and others have previously shown that the ICR controlling *Dlk1-Dio3*, called the intergenic differentially methylated region (IG-DMR), frequently becomes hypermethylated in a context-dependent manner during *in vitro* reprogramming of somatic cells (Carey et al., 2011; Stadtfeld et al., 2010). This yields induced pluripotent stem cells (iPSCs) with LOI and upregulation of *Dlk1* that is indicative of expression from both alleles without paternal bias (Stadtfeld et al., 2010). Together, these observations suggest that the insertion of fluorescent reporter genes into the endogenous *Dlk1* locus might enable novel approaches to study imprinted gene expression in living cells and tissues.

## RESULTS AND DISCUSSION

### A reporter model for allele-specific expression of *Dlk1*

**Fig. 1.**
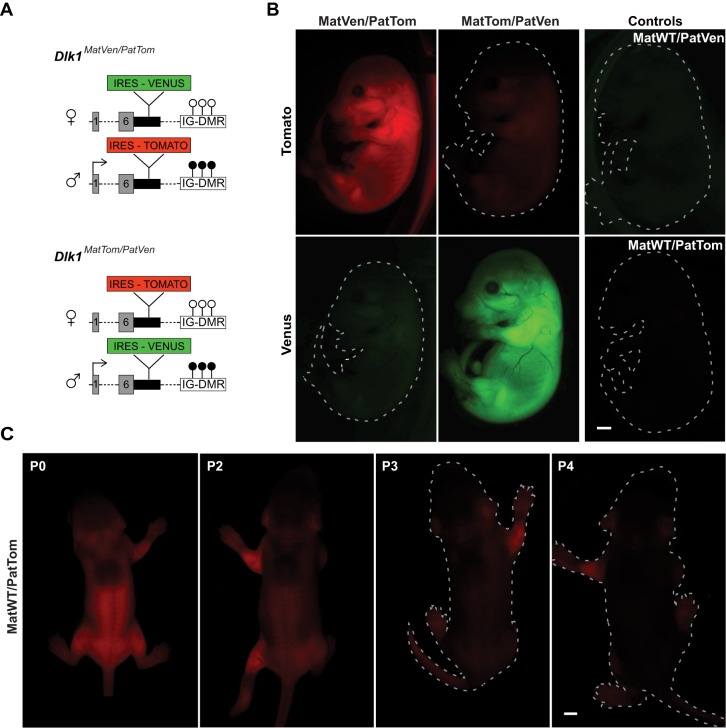
**Reporter knock-in alleles capture imprinted *Dlk1* expression *in vivo*.** (A) Genetic configuration of *Dlk1* reporter mice with coding sequences of Tomato (Tom) and Venus (Ven) inserted into the paternal (Pat) and maternal (Mat) allele of *Dlk1*: *Dlk1^MatVen/PatTom^* (top panel) or *Dlk1^MatTom/PatVen^* (bottom panel). Circles indicate methylation status of the IG-DMR and an arrow at exon 1 indicates imprinted expression from the paternal allele. (B) Fluorescent images of E16.5 mouse embryos of the corresponding genotypes. (C) Whole body images of *Dlk1^MatWT/PatTom^* mice at postnatal day (P)0 to P4. Scale bars: 1 mm (B) and 2 mm (C).

To generate a mouse reporter system for imprinted *Dlk1* expression, we inserted the coding sequence for the green/yellow fluorescent protein Venus or the red fluorescent protein tdTomato (Tomato), into the three prime untranslated region (3′UTR) of endogenous *Dlk1* (Fig. 1A). In accordance with widespread *Dlk1* expression at perinatal stages (da Rocha et al., 2007), embryonic day (E)16.5 mice that had inherited reporter alleles from the father exhibited strong fluorescence (Fig. 1B). Reporter gene expression from the maternal allele was greatly diminished but detectable above background levels (Fig. 1B). The brightness of the paternal reporter allele allowed direct visualization of *Dlk1* expression in living pups. This revealed expression in the growing limbs and the trunk until postnatal day (P)2, when it rapidly declined and became nearly undetectable by P4 (Fig. 1C). These findings are in accordance with previous studies (Lui et al., 2008), indicating that our reporter model recapitulates expression hallmarks of *Dlk1*.

### Identification of organ-specific expression patterns at the single-cell level

**Fig. 2.**
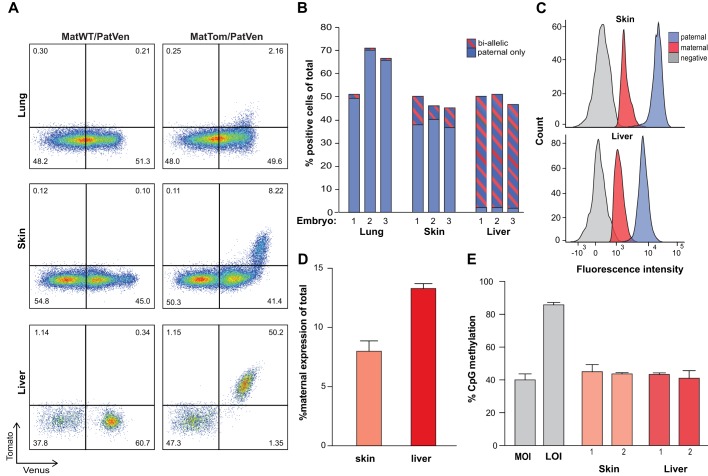
**Quantification of allele-specific expression in developing tissues.** (A) Flow cytometric analysis of fetal tissues from *Dlk1^MatWT/PatVen^* (left column) control mice or *Dlk1^MatTom/PatVen^* (right column) dual-reporter mice at E16.5. (B) Percentage of *Dlk1^+^* cells with paternal-only and bi-allelic expression in fetal lung, skin and liver. (C) Mean fluorescent intensity of paternal and maternal *Dlk1^Tom^* in bi-allelic fetal skin and liver cells. (D) Quantification of relative strength of maternal to total *Dlk1* in bi-allelic cells as measured by flow cytometry. Error bars indicate s.e. (*n*=3 embryos). (E) DNA methylation at the IG-DMR in FACS-sorted fetal skin and liver cells with active maternal *Dlk1* allele from two embryos. Methylation levels in iPSCs with established LOI or MOI were used as controls. Error bars indicate s.e. (*n*=28 CpGs analyzed).

We next sought to test whether the dual-reporter system could provide insight into allele-specific *Dlk1* expression during development. Therefore, we analyzed E16.5 lung, skin and liver, representing tissues for which a developmental function of *Dlk1* has been reported (Driskell et al., 2013; Tanimizu et al., 2003; Weng et al., 2009; Wu et al., 2008). Flow cytometric analysis revealed strong paternal reporter expression and detectable levels of maternal expression in some cells (Fig. 2A). The number of such bi-allelic cells ranged from rare (∼4% of Dlk1^+^ cells) in lung, to predominant (>95%) in liver (Fig. 2A,B). Importantly, the reporter insertions did not alter the expression levels of *Dlk1* or the reciprocally expressed *Gtl2* gene in the tissues analyzed (Fig. S1). Of note, while the reporter alleles revealed relatively homogenous expression profiles in liver and lung, two distinct populations could be defined based on allele-specific *Dlk1* expression in skin: one with paternal and one with bi-allelic expression (Fig. 2A). This expression dichotomy has not previously been identified (Driskell et al., 2013) and suggests that the dual reporter could aid in the identification of novel cell populations. Comparison of reporter activity in B6 background and B6×129 F1 mice revealed similar expression patterns, but subtle differences in the abundance of expressing cells, suggesting that the model might be useful to study strain-specific aspects of *Dlk1* regulation (Fig. S2).

We did not observe cells expressing only maternal *Dlk1* within the analyzed tissues (Fig. 2A) and the intensity of the maternal reporter in bi-allelic cells remained comparatively weak in both reporter configurations (Fig. 2C; Fig. S3). Quantification of fluorescence intensity suggested that maternal expression levels in the skin and liver range between 7 and 14% of total *Dlk1* expression within the bi-allelic cell populations (Fig. 2D). This observation suggests relaxation of imprinting (ROI), defined by incomplete silencing of the maternal allele, rather than LOI, as the paternal expression bias is still apparent. In support of this conclusion, we did not detect elevated levels of DNA methylation at the IG-DMR in skin or liver cells isolated based on maternal *Dlk1* expression (Fig. 2E). Overall, the pattern of *Dlk1* reporter activity is consistent with studies that report maternal expression in liver but fail to detect it in lung (da Rocha et al., 2007; Sato et al., 2011). Analysis of allele-specific *Dlk1* expression by quantitative PCR in whole tissue confirmed that the activity of the maternal allele is not altered by the reporter insertion (Fig. S4). These results suggest that the *Dlk1* reporter model allows sensitive and reliable detection of tissue-specific expression patterns.

### Detection of loss of imprinting upon cellular reprogramming

**Fig. 3.**
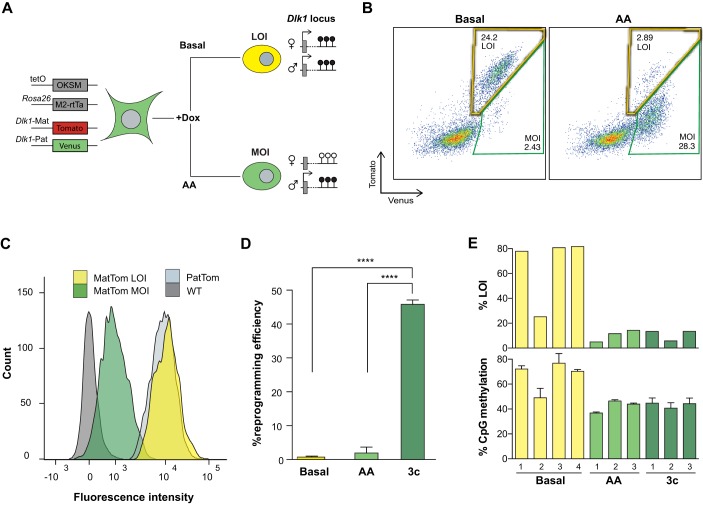
**Detection of LOI induced by cellular reprogramming.** (A) Transgenic alleles in fibroblasts isolated from reprogrammable imprinting reporter animals. The expected behavior of the reporter genes in cells derived upon reprogramming in basal or AA conditions is illustrated. (B) Representative FACS plots of RA-differentiated iPSCs derived via basal reprogramming (left) or in AA conditions (right). Yellow and green gates indicate LOI- and MOI-expressing cells, respectively. (C) Mean fluorescence intensity of Tomato in the indicated genotypes. (D) Reprogramming efficiency measured as percentage of stable iPSC colonies per input MEFs in basal conditions, AA or 3c. ****P<0.00005 with a one-way ANOVA and Tukey's multiple comparison test. Error bars indicate standard error (*n*=3). (E) LOI-level *Dlk1* expression (top panel) and DNA methylation at the IG-DMR (bottom panel) in iPSC cultures derived in the indicated conditions.

Next, we set out to test whether the reporter system can faithfully detect instances of bona fide LOI. For this, we took advantage of the observation that reprogramming of murine fibroblasts by the transcription factors, Oct4, Klf4, Sox2 and Myc (together referred to as OKSM), frequently yields induced pluripotent stem cells (iPSCs) with DNA hypermethylation of the IG-DMR and upregulation of *Dlk1* (Liu et al., 2010; Stadtfeld et al., 2010). This abnormality results in reduced developmental potential but can be prevented by addition of ascorbic acid (AA) to the reprogramming medium (Stadtfeld et al., 2012). We therefore anticipated that reprogramming of *Dlk1* reporter fibroblasts in basal conditions would predominantly yield cells with equal paternal and maternal expression, while reprogramming in the presence of AA would prevent the occurrence of such cells (Fig. 3A).

In agreement with low levels of *Dlk1* transcription in pluripotent cells (Kota et al., 2014), no reporter gene expression could be detected in naïve iPSCs. However, exposure to the differentiation-inducing agent retinoic acid (RA) yielded cells with readily detectable reporter gene fluorescence. As shown in Fig. 3B, cells derived in basal conditions almost exclusively expressed both reporter alleles, whereas monoallelic fluorescence was prevalent in cells obtained in the presence of AA. In contrast to our observations in the embryo, paternal and maternal expression levels in cells generated in basal reprogramming conditions were indistinguishable (compare Fig. 2C and Fig. 3C). As this is consistent with imprinting loss, we will refer to such cells as ‘LOI’ cells, while we call those with paternal-only or bi-allelic with low maternal expression ‘MOI’ cells (see Fig. 3B). In agreement with this categorization, iPSCs with a high degree of LOI expression showed strongly elevated levels of DNA methylation at the IG-DMR (Fig. 3E; Fig. S5).

These observations suggest that the *Dlk1* reporters accurately reflect changes in imprinting status during reprogramming. We also evaluated *Dlk1* imprinting in iPSCs derived by combined modulation of TGFβ and Wnt signaling in the presence of AA (referred to as ‘3c’ conditions) (Vidal et al., 2014), which increases reprogramming efficiencies 10- to 20-fold (Fig. 3D). We found that differentiation of iPSCs derived in 3c mirrored the results obtained in AA conditions, with the majority of cells exhibiting normal DNA methylation levels and MOI expression of *Dlk1* (Fig. 3E; Figs S5,S6). This suggests that this highly efficient reprogramming condition might provide a tractable method to study factors that contribute to imprinting maintenance.

### Modulation of allele-specific *Dlk1* expression by oxygen levels during iPSC culture and differentiation

**Fig. 4.**
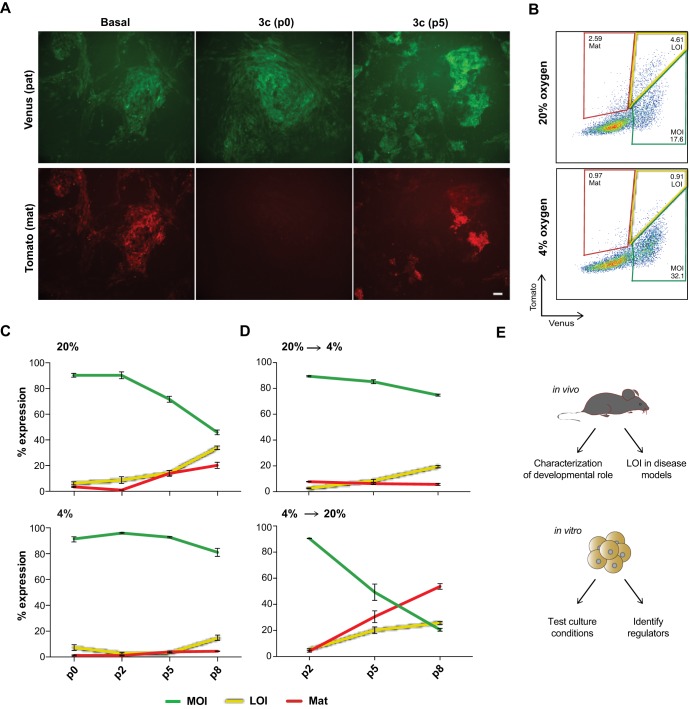
**Instability of allele-specific expression during culture and *in vitro* differentiation of pluripotent cells.** (A) Allele-specific reporter expression in differentiating cultures of early passage *Dlk1^MatTom/PatVen^* iPSCs derived in basal conditions (left column) or iPSCs derived in 3c at either early (P0, middle column) or higher (P5, third column) passage. Scale bar: 100 µm. (B) Gating strategy to define cells based on their *Dlk1* expression pattern in 3c iPSCs maintained and differentiated in 4% or 20% oxygen; P5 cultures are shown. Red gates indicate maternal-only expression (mat), yellow gates show LOI expression and green gates indicate MOI. (C) Relative abundance of MOI (green), LOI (yellow) or maternal-only (red) cells in cultures expanded and differentiated at the indicated passage number in either 20% (top) or 4% (bottom) oxygen. (D) Same samples as in C but iPSCs expanded in 20% oxygen were differentiated in 4% oxygen (top panel) and iPSCs expanded in 4% oxygen were differentiated in 20% oxygen (bottom panel). Error bars indicate s.e. (*n*=3). (E) Possible applications of the *Dlk1* reporter model to study genomic imprinting.

Despite the striking difference in maternal reporter expression between cells derived in basal and 3c conditions, we noticed that differentiation of AA and 3c iPSCs frequently yielded a small subset of LOI cells (Fig. 3B). Such cells were rare when differentiating freshly generated iPSCs but became more frequent with iPSCs at higher passage, along with a number of cells with maternal-only expression (Fig. 4A). All *in vitro* experiments described thus far were conducted in standard culture conditions, including 20% oxygen levels. In light of the reported impact of molecular oxygen on epigenetic processes in pluripotent cells, including imprinting (Xie et al., 2014) and X-chromosome inactivation (Lengner et al., 2010), we tested the impact of oxygen concentration on allele-specific *Dlk1* expression. Taking advantage of the 3c system, which enables analysis at earlier passage, we compared iPSCs that were expanded and differentiated in atmospheric (20%) and physiological (4%) oxygen levels. We observed no significant difference with early passage iPSCs, but a gradual increase in cells with LOI and maternal-only reporter expression in 20% oxygen conditions at higher passage (Fig. 4B,C). In contrast, predominantly MOI expression was maintained in 4% oxygen (Fig. 4B,C). When cells expanded in 20% oxygen were differentiated in 4% oxygen, the frequency of cells with LOI and maternal-only expression remained low, while their numbers strongly increased in the reverse conditions (expansion in 4%, followed by differentiation in 20% oxygen) (Fig. 4C; Fig. S7). This indicates an unexpected susceptibility of the maternal *Dlk1* allele to become reactivated upon prolonged expansion and differentiation of iPSCs at elevated oxygen levels. Because imprint dysregulation has been recognized as a concern for the quality of pluripotent cell lines (Greenberg and Bourc'his, 2015), we anticipate that the *Dlk1* reporter system will be useful in optimizing stem cell derivation and culture conditions.

In summary, we have developed a reporter model that serves as a sensitive indicator for allele-specific expression of *Dlk1* and allows prospective isolation of even small subsets of cells with paternal, bi-allelic, LOI or maternal-only expression patterns. Our observations are consistent with tight control of *Dlk1* imprinting during early developmental stages in the tissues analyzed, but suggest frequent erosion during *in vitro* manipulations. The system described here should therefore provide a novel means to systematically study *Dlk1-Dio3* regulation in diseased tissues, pluripotent cell culture and during development (Fig. 4E). This should aid in refining our understanding of the molecular processes involved in the establishment and maintenance of imprinting.

## MATERIALS AND METHODS

### Transgenic mice

All animals were on a B6 background, unless otherwise indicated. For reprogramming experiments, *Dlk1* reporter mice were crossed with animals carrying an inducible OKSM transgene (Stadtfeld et al., 2010). *Dlk1* reporter mice were generated as described in the supplementary Materials and Methods. All animal experiments were done in accordance with the guidelines of the NYU School of Medicine IACUC.

### Cell culture and reprogramming

Pluripotent cell culture and MEF reprogramming was conducted using previously described procedures (Vidal et al., 2014) with modifications as described in the supplementary Materials and Methods. Cells were reprogrammed for 12 days (basal conditions), 10 days (AA) or 6 days (3c) in the same oxygen condition in which they were later expanded (4% or 20%).

### Cell differentiation and flow cytometry

Trypsinized iPSCs were pre-plated for 30 min to remove feeder cells and seeded onto gelatinized plates at a density of 30,000 cells/cm^2^. The next day, fibroblast medium with 0.4 µg/ml retinoic acid was added, followed by daily media changes and imaging using a Nikon Eclipse TiE inverted microscope with filters to detect Venus (excitation, 500/20 nm; emission, 535/30 nm) and Tomato (excitation, 545/30 nm; emission, 620/60 nm). For quantification of *Dlk1* reporter expression, dissociated cultures were acquired on an LSRII cytometer (BD Biosciences) and analyzed with FlowJo software (Tree Star).

### Tissue isolation and reporter detection

Mouse embryos isolated at E16.5 were imaged using a Nikon SMZ1500 Stereo Fluorescence Microscope. For flow cytometry, isolated tissues were incubated for 30 min at 37°C in 0.25% trypsin. During this time, tissues were disassociated using progressively smaller pipet tips (1000 μl to 200 μl), followed by analysis on an LSRII or sorting on a FACSAria (BD Biosciences).

### DNA methylation analysis

Genomic DNA was isolated using proteinase K in lysis buffer, pH 8 (100 mM Tris-HCl, 5 mM EDTA, 0.2% SDS, 200 mM NaCl) and reconstituted in TE buffer, pH 7.5 (10 mM Tris-HCl, 1 mM EDTA). DNA pyrosequencing and next-generation bisulfite sequencing was conducted using the ADS1452 assay, which covers the IG-DMR (chr.12:109,528,253-109,528,471 in mm10) (EpigenDX).

### Gene expression analysis

RNA isolated using the miRNeasy kit (Qiagen) was used for cDNA preparation with the Transcriptor HIFI cDNA synthesis kit (Roche). Samples were run on a LightCycler 480 Real-Time PCR System (Roche). Relative allele-specific expression was assessed using qRT-PCR with primers as described in the supplementary Materials and Methods.
